# Obstetrics and Gynecology

**Published:** 1899-11

**Authors:** William Warren Potter

**Affiliations:** Buffalo, N. Y.


### OBSTETRICS AND GYNECOLOGY.

Conducted by WILLIAM WARREN POTTER, M. D., Buffalo, N. Y.

### URINARY TOXICITY IN PREGNANT WOMEN.

Labadie-Lagrave and others (*Archives génerales de Médecine*, May 1899; *University Medical Magazine*) state at the beginning of their article that hertofore the association of urinary toxicity with pregnancy has had reference only to eclampsia; and, that, therefore, there should be instituted a special study of the intoxicating properties of the urine of the pregnant woman without special reference to eclampsia or any other single condition. It should, however, be stated that studies of general scope have been made in this direction for the latter months of pregnancy. The present authors have studied the question from the beginning of gestation, with a view of determining when the urine becomes toxic.

With regard to the first six weeks after impregnation, or rather after the last menstrual period, the normal toxicity undergoes a diminution instead of an increase (which might theoretically have been anticipated); it would therefore appear that this diminution of toxicity may serve as an early sign of pregnancy. If a suspected case of pregnancy exhibits normal toxicity, we may therefore reckon upon the existence of hepatic insufficiency; provided pregnancy is actually present.

If, however, the urinary toxicity is diminished, we cannot at once jump to the conclusion that the woman is pregnant, because this diminution of toxicity is also present in chlorosis, hysteria and tuberculosis. A chlorotic subject undergoes rapid recovery of normal toxicity under appropriate treatment; but if such a patient becomes pregnant the toxicity will not increase under the customary treatment. This is also true of the hysterical and tubercular subject. If patients of this sort do not improve under suitable treatment and have been exposed to impregnation the failure to regain the normal toxicity is readily explained.

The authors show by a toxicity curve that a given patient had her toxicity lowered in the third month, slightly recovered in the fourth, lowered again in the fifth, partly regained in the sixth, practically unchanged until delivery, the normal toxicity being regained in some six weeks after delivery.

The authors therefore conclude that normally, there should occur a marked lowering of urinary toxicity in pregnancy, and thus whenever this law is violated there is always a definite reason for it, and that if a pregnant woman has a toxicity equal or superior to normal, we should fear the possible development of eclampsia.

---

### CAUSES OF STERILITY.

LEFÈVRE (*La Gynécologie*, August, 1899; *British Medical Journal*) dwells on certain complex causes of sterility. A woman may be barren owing to a single definite pathological condition. Very frequently, however, it is quite otherwise. In eighty-four cases of sterility, observed by Doléris and Lefèvre, several pathological conditions, none of themselves sufficient to prevent conception, were detected in combination in sixty-six. This group of factors together effective in maintaining sterility consists of catarrh of the cervix, conical cervix, stenosis of the os externum, acute anteflexion, and free prolapse of the posterior vaginal wall. Hence treatment should be directed to all these complications, else failure will be certain. The endocervicitis will require the curette, or if the mucosa show intractable changes, amputation of the cervix, after Schröder's method. That operation is also required when the cervix is conical or stenosed, so as to furnish the patient with an os externum wide enough to admit semen. Acute anteflexion is best met by frequent dilatation. If it tend to return, a stem pessary, a colporrhaphy in front of the cervix will be required, whilst posterior colpocele will demand retrocervical colporrhaphy; forty-nine of the sixty-six cases were treated on these principles. In eight the treatment clearly failed. In ten pregnancy has not yet occurred, but either the after-history was too short or there were other factors present accounting for sterility, such as impending menopause (2), uterine fibroid (2), great obesity (1), and the like. There remained twenty-four cases where success was evident, nineteen patients having borne one child, three patients two children, and two patients three children after treatment.

